# Effectiveness of perineural administration of dexamethasone with lidocaine on onset time of sensory block and early postoperative analgesia in axillary brachial plexus block: a prospective cohort study, Ethiopia

**DOI:** 10.1097/MS9.0000000000001741

**Published:** 2024-01-22

**Authors:** Simeneh Mola, Betelihem Girma

**Affiliations:** a Department of Anesthesia, Dilla University, College of Medicine and Health Science, Dilla; b Department of Anesthesia, Addis Ababa University, College of Health Science, Addis Ababa, Ethiopia

**Keywords:** adrenaline, Brachial plexus block, lidocaine, perineural dexamethasone, postoperative analgesia

## Abstract

**Introduction:**

The axillary brachial plexus block is a popular nerve block for forearm, wrist, and hand surgery. The aim of this study was to assess the effectiveness of perineural administration of dexamethasone as an adjunct to lidocaine with adrenaline on the onset of sensory block and early postoperative analgesia in trans-arterial axillary brachial plexus block.

**Methodology:**

This single-centered prospective cohort study recruited 68 adult patients, 34 in each groups. The frequently used 8 mg dexamethasone combined with 1% lidocaine and adrenaline was investigated. The normality of the data was checked using the Shapiro–Wilk test. An independent *t*-test was used to compare the mean values of symmetric numeric data. Categorical variables between the two groups were analyzed using χ^2^. The Mann–Whitney *U* test and Kaplan–Meier method using the log-rank test were used to compare asymmetric numeric data, and a *P*-value of <0.05 was considered as significant.

**Results:**

The median onset time of sensory block was comparable between the nonexposed (24(6) min) and exposed group (24(6) min) (*P*=0.068). However, the duration of sensory block was significantly longer in the exposed group (235.5±37.51 min) than the nonexposed group (172.76±28.19 min) (*P*<0.001). The time to the first analgesic request was significantly longer in the exposed than the nonexposed group (*P*<0.01). Postoperative pain scores were significantly lower at 4 and 8 h in the exposed group (*P*<0.05).

**Conclusion and recommendations:**

The addition of 8 mg dexamethasone to 1% lidocaine with adrenaline solution in trans-arterial axillary brachial plexus block for ambulatory elective hand, wrist, and forearm surgeries prolonged the duration of sensory blockade and the first analgesic request time but did not reduce the onset time of sensory block. The authors recommend the addition of 8 mg dexamethasone to 1% lidocaine with adrenaline solution to prolong the duration of sensory block and the first analgesic request time.

## Introduction

Highlights

- The duration of sensory blocked was significantly longer with perineural dexamethasone as an adjunct to trans-arterial axillary brachial plexus block.
- Perineural addition of dexamethasone prolonged analgesia request time.
- The time for the first analgesia request was longer with perineural dexamethasone with lidocane.
- A significant reduction in the severity of postoperative pain scores was seen at 4 and 8 h after incision in the dexamethasone group.

Hand surgery is a specialty that employs combined skills from the overlapping specialties of orthopedic surgery, plastic surgery, and emergency medicine. Regional anesthesia is an excellent adjunct to general anesthesia for hand and wrist surgery. It provides superior postoperative analgesia and hastens recovery from anesthesia^1^. Surgery for the upper extremity, from the shoulder to the hand, can be performed successfully by blocking the brachial plexus at several points until it branches into peripheral nerves^2^.

The axillary brachial plexus block is a popular nerve block for forearm, wrist, and hand surgery^3^. It is useful as both sole anesthesia and a supplement to general anesthesia and provides effective postoperative analgesia, reducing the need for opioid analgesics^4,5^.

The axillary approach to brachial plexus blockade has the advantage of being performed away from the pleura and neuraxial structures, so it is ideal to obtain a block with a minimum of discomfort, complications, and side effects^6^.

There are few contraindications to axillary brachial plexus blocks. Local infection, neuropathy, and bleeding risk must be considered^7^. Nerve stimulation, ultrasound, paresthesia, or trans-arterial technique may be used to perform an axillary brachial plexus block based on the availability of resources, skills, and the preferences of the anesthesia provider. The trans-arterial technique is a widely accepted technique in resource-limited countries. It is relatively easy to perform, does not require sophisticated and expensive materials like nerve stimulators and ultrasound. However, it is associated with a higher incidence of systemic toxicity and has a lower success rate than other techniques^7,8^.

Dexamethasone is a high-potency, long-acting glucocorticoid with little mineralocorticoid effect that has been used for prophylaxis of postoperative nausea. Single doses of dexamethasone have also been reported to improve analgesia after various operations, whether by oral, intravenous (I.V.) or perineural route^9^. Recent evidence demonstrates a potential role of dexamethasone in postoperative pain management, both as a systemic analgesic and as an adjunct to local anesthetics for perineural use.

The aim of this study was to assess the effectiveness of perineural administration of dexamethasone as an adjunct to lidocaine with adrenaline on the onset of sensory block and early postoperative analgesia in the trans-arterial axillary brachial plexus block for ambulatory elective forearm, wrist, and hand surgery.

## Methods and materials

### Study setting and participants

An exposure-based prospective single-centered cohort study was conducted from 1 January to 1 June 2018 at one of the government-owned hospitals located in Addis Ababa, the capital city of Ethiopia. Ethical clearance was obtained from the institutional review board. Verbal and written informed consent was obtained from each participant, and copies of the written consent are available on request. All American Society of Anesthesiologist (ASA) I and ASA II patients aged 18–60 year scheduled for elective forearm, wrist, and hand surgery under tans-arterial axillary brachial plexus block were included while duration of surgery less than half hour or greater than 2 hours, BMI >35 kg/m², chronic opioid use, failed axillary block, users of local anesthetics other than 1% lidocaine with adrenaline, users of other adjuvants, patients who took sedative or analgesics premedication within 24 h preoperatively, bleeding abnormality, known allergy to local anesthetics, history of diabetics mellitus, history of peptic ulcer, septic patients, peripheral neuropathy, or nerve injuries were excluded.

The primary outcome variables were onset and duration of sensory block; the secondary outcome variables were first analgesia request time and severity of postoperative pain measured by a numeric rating scale.

#### Sample size determination and sampling procedure

The required sample size was obtained using two independent sample size formula based on the mean difference of onset of sensory block, postoperative numeric rating scale (NRS) score, time to the first analgesia request, and duration of sensory block from the previous study done in Iran^10^. The mean onset of sensory block was 14±5 min in the control group and 11±4 min in the dexamethasone group, with a CI of 95% and a power of 80%. Four patients were lost to follow-up during the study, and a total of 68 patients were enrolled, with 34 patients in each group.

A systematic random sampling method was used for data collection. To assure the reliability of the data a pretested questionnaire was used and training was given to data collectors. Socio-demographic data and baseline status were filled out from the patient chart. The onset time of sensory blockade of each nerve was assessed every 3 min by pinprick stimulation on the corresponding cutaneous innervation of the nerves and compared with the same stimulation on the contra-lateral hand on all branch of the brachial plexus. Duration of sensory block was assessed every 20 min, while severity of pain using NRS was assessed at 2, 4, 6, and 8 h after incision staring time.

#### Data analysis and interpretations

The data was analyzed using SPSS version 20. The Shapiro–Wilk test was used to test for distributions of data and presented in terms of mean±SD for symmetric data and median (interquartile range) for asymmetric numeric data. Homogeneity of variance was assessed using Levene’s test for equality of variance. The comparison of numerical variables between the two study groups was done using an independent *t*-test for normally distributed data and a Mann–Whitney *U* test for skewed numerical data. The first analgesic request was analyzed by the Kaplan–Meier survival analysis with the log-rank test. Frequency and percentage were used to describe categorical variables and statistical differences between the groups were tested using the χ^2^ and Fisher exact test as required. A *P*-value <0.05 was considered statistically significant.

This study was reported in line with the strengthening the reporting of cohort, cross-sectional, and case–control studies in surgery (STROCSS) guideline^11^.

## Results

### Socio-demographic characteristics of study participants

There was a comparable distribution of socio-demographic and surgery-related variables between the groups. There was no statistically significant difference between groups regarding baseline hemodynamic variables and numeric rating scale scores (Table 1).

**Table 1:** Demographic and perioperative characteristics of patients

Variable	Nonexposed groups (*n*=34)	Exposed groups (*n*=34)	Total	*P*
Sex^a^				
Male	23 (48.9%)	24 (51.1%)	47 (100%)	0.793
Female	11 (52.4%)	10 (47.6%)	21 (100%)	
Age^b^	33.44±11.01	34.29±8.15		0.718
ASA status^a^				
ASA I	25 (47.2%)	28 (52.8%)	53 (100%)	0.384
ASA II	9 (60%)	6 (40%)	15 (100%)	
BMI^b^	22.01±2.47	22.62±1.73		0.244
Baseline heart rate (bpm)^b^	72.56±4.59	74.35±5.08		0.132
Baseline SBP (mmHg)^b^	124.79±9.48	121.91±8.02		0.181
Baseline DBP (mmHg)^c^	80 (14)	75.5 (10)		0.144
Baseline NRS^c^	1 (2)	1 (2)		0.267
Volume of local anesthetics (ml)^c^	40 (5)	35 (5)		0.267
Duration of surgery (minutes)^b^	83.62±16.97	86.29±12.90		0.467

a Frequency (percentage), χ^2^ test used.

b Values presented as mean±SD, independent *t*-test was used for analysis.

c Median (IQR), U and make it Mann-Whitney U test used.

### Onset time of sensory block

There was a statically insignificant difference in the onset time of complete sensory block between the exposed group median (IQR) of 24 (6) min and the nonexposed group 24(6) min with (*P*=0.068) (Table 2).

**Table 2:** Comparison of onset time of sensory block and incision starting time between the two groups

Variables	Nonexposed group (*n*=34)	Exposed group (*n*=34)	*P*
Onset time of complete sensory block (minutes)^a^	24 (6)	24 (6)	0.068
Incision starting time (minutes)^b^	33.56±4.12	32.22±4.23	0.203

a Values presented as Median (IQR) Mann–Whitney *U* test was used for analysis.

b Values presented as mean±SD (independent *t*-test was used for the analysis).

*P*<0.05 was considered as significant.

**Figure 1:**
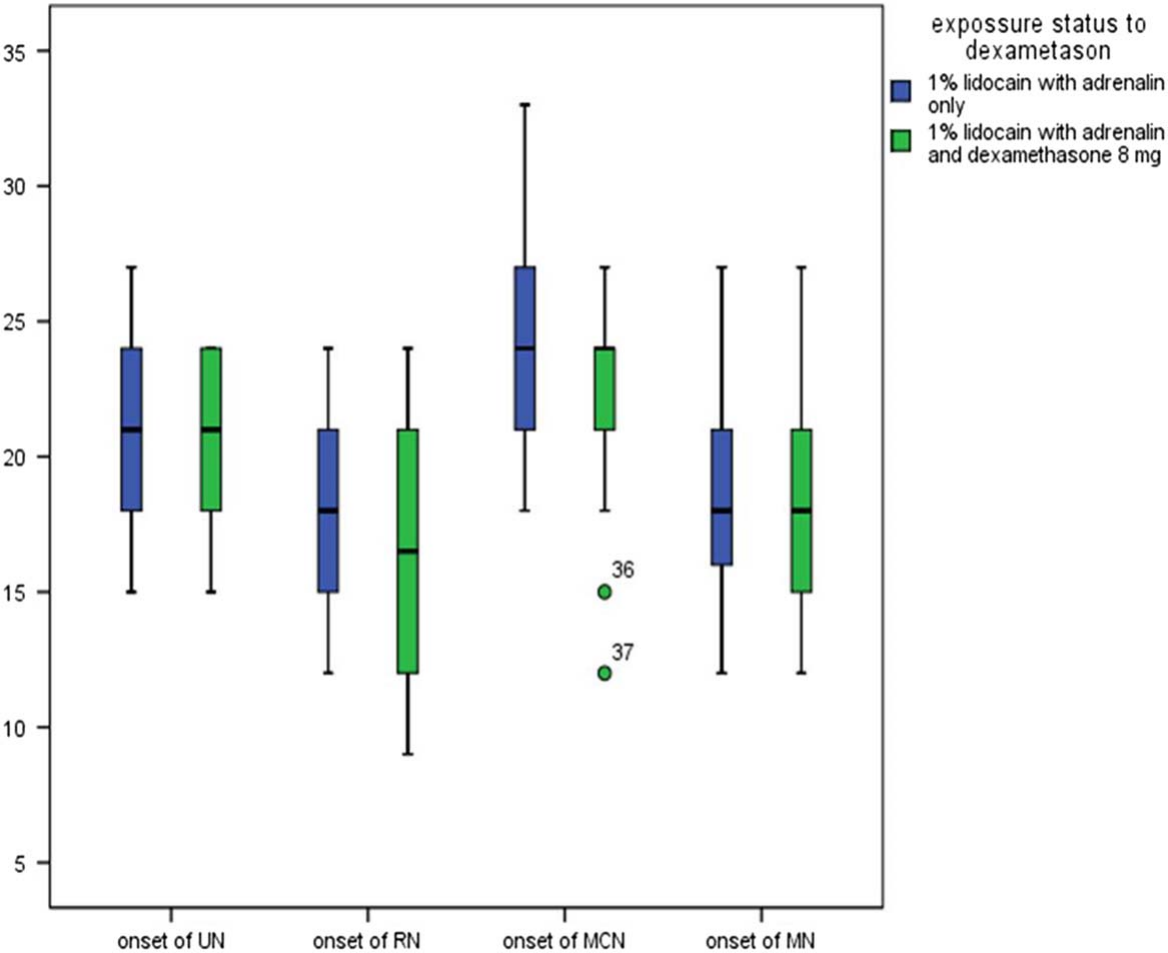
Comparison of median onset time of analgesia (in minutes) for all nerves of axillary block Mann–Whitney *U* test was used for analysis. MN, median nerve; MCN, musculo cutaneous nerve; RN, radial nerve; UN, ulnar nerve.

The onset time of sensory block for the four major branches of the brachial plexus (ulnar, radial, musculocutaneous, and median) was analyzed by the Mann–Whitney test and there was no significant difference observed between the groups (Fig. 1).

### Duration of sensory block

**Figure 2:**
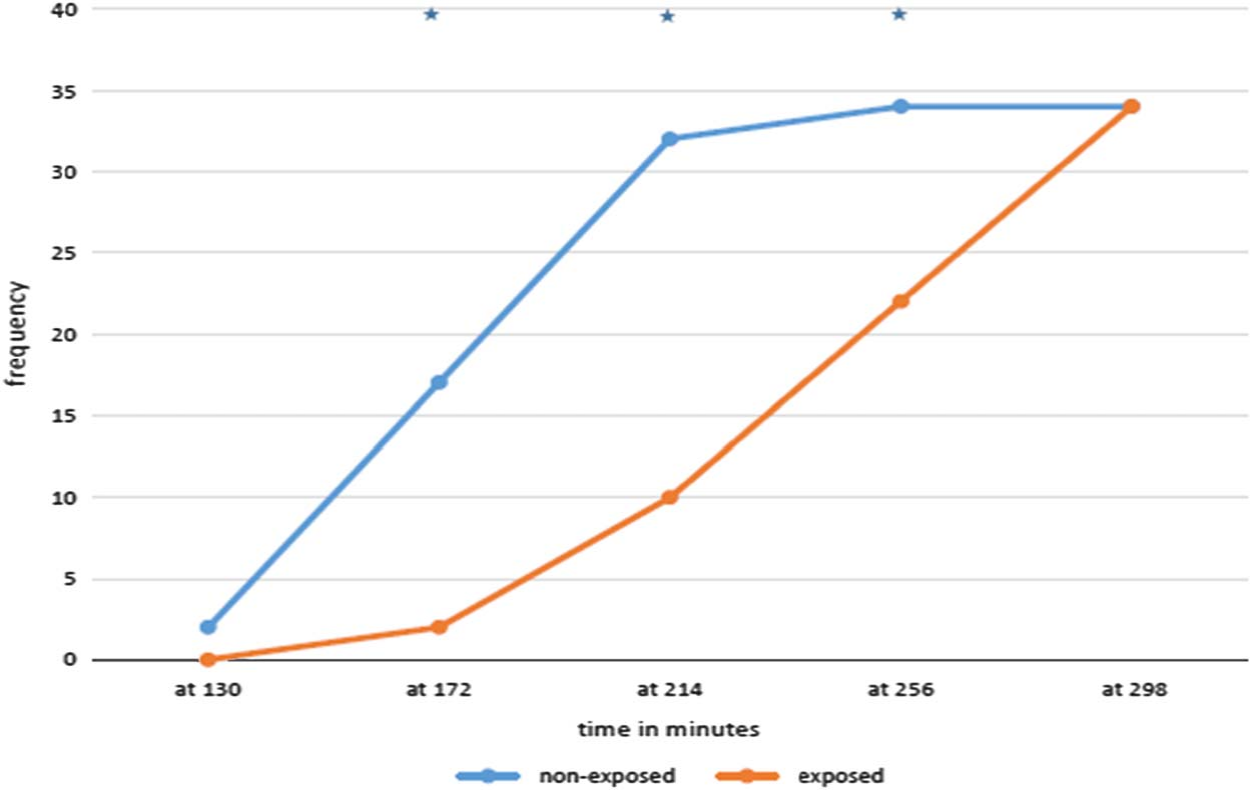
Comparison of cumulative frequency of patients recovered from analgesia at a given time between the groups, analyzed using the Fisher exact test at 130 min and the χ^2^ test at 172, 214, 256, and 298 min. * indicate *P*<0.05.

The duration of sensory blockade was significantly longer in the exposed group than in the nonexposed group. The mean duration of sensory block in the nonexposed group was 172.76±28.19 min, while in the exposed group it was 235.5±37.51 min, with *P*<0.001. The analysis of the cumulative distribution of patients recovered from analgesia using the Fisher exact test at 130 min and the χ^2^ test at 172, 214, 256, and 298 min showed a significant difference between the groups at 172, 214, and 256 min (Fig. 2).

### Time to first analgesia request

**Figure 3:**
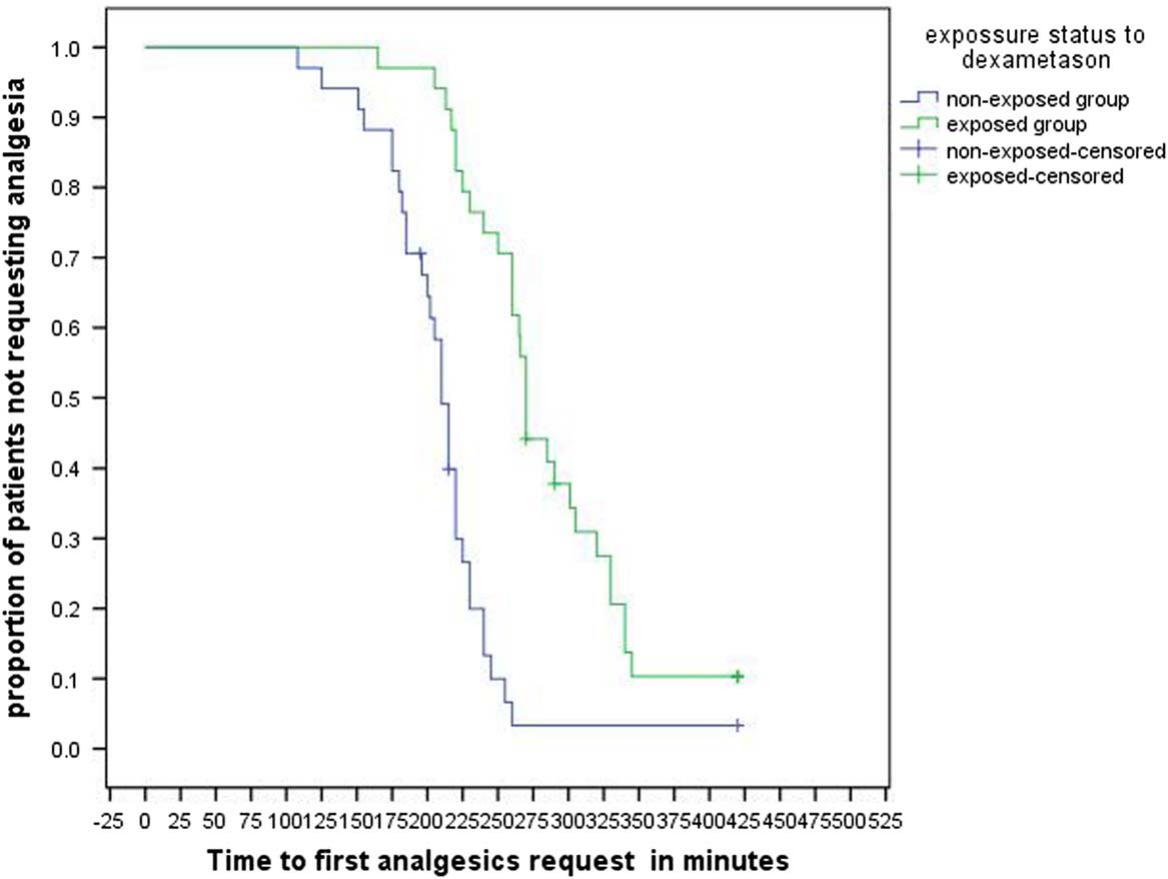
Kaplan–Meier curves (log-rank test) for the first analgesic request (*P*<0.001).

The Kaplan–Meier curve was used for the first analgesic request. The first analgesic request considered as an event and patients not receiving any analgesics 7 h after local anesthetics injection and lost to follow-up were censored to the right as presented in Figure 3. Significant differences between these curves (log-rank test) were obtained between exposed and nonexposed groups (*P*<0.001). The median (IQR) time to first anesthesia request was 210 (185 to 230) minutes in the nonexposed group and 270 (240 to 330) minutes in the exposed group (Fig. 3).

### Early postoperative pain score

The median (IQR) NRS scores assessed between groups at 2, 4, 6, and 8 h starting from the incision showed a significant difference in pain severity between the groups at 4 and 8 h (Table 3).

**Table 3:** Comparison of postoperative pain severity using 11 point NRS score (0–10) between the two groups

	Nonexposed group (*n*=34)	Exposed group (*n*=34)	*P*
Two hour after incision in NRS	0 (0)	0 (0)	0.79
Four hour after incision in NRS	5 (3)	2 (3)	<0.001
Six hour after incision in NRS	4 (2)	4 (2)	0.289
Eight hour after incision in NRS	3 (2)	1 (1)	<0.001

Values were presented as median (interquartile range), and Mann–Whitney *U* test was used for analysis, *P*<0.05 considered as significant.

### Postoperative nausea

**Figure 4:**
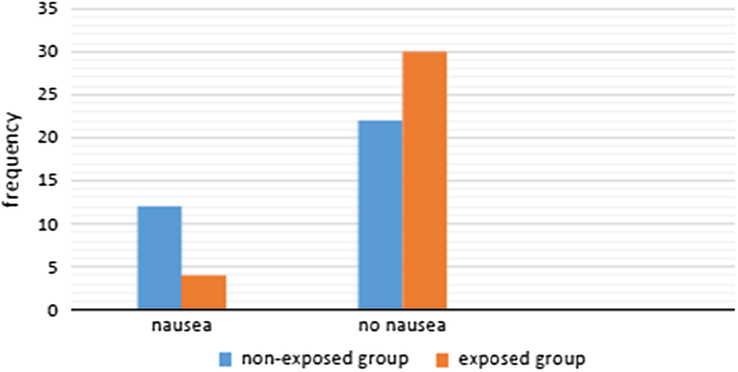
Comparison of incidence of nausea and vomiting between the two groups.

There was a significant difference in the incidence of postoperative nausea at 6 h between the nonexposed group 12 (35.3%) and the exposed group 4 (11.8%), *P*=0.045 but there was no significant difference on the incidence of vomiting between the groups (Fig. 4).

## Discussion

The mechanism of dexamethasone prolonging the duration of nerve blocks is not completely understood and is thought to arise from various factors such as vasoconstriction produced by steroids that decrease the absorption of local anesthetics, its inhibitory action on potassium channels on pain sensory nerves, the anti-inflammatory action of dexamethasone and blocking transmission of nociceptive C fibers^12,13^.

Currently, many studies have compared various doses of perineural dexamethasone such as 1 mg, 2 mg, 4 mg, 5 mg, and 8 mg but the results remain controversial^14,15^. A systematic review on the safety and efficacy of perineural dexamethasone as an adjunct for peripheral nerve blockade in 29 controlled trials with 1695 participants showed the addition of perineural dexamethasone as an adjuvant to short, medium, and long‐duration action local anesthetics prolonged the postoperative analgesia and motor blockade but the evaluation of the relationship between the different doses of dexamethasone and duration of analgesia remained inconclusive^16^.

The results of the current study revealed that the addition of 8 mg dexamethasone to 1% lidocaine with adrenaline prolonged the duration of sensory block but did not speed up the onset time of complete sensory block. The median time to onset of complete sensory block was similar in the exposed group 24(6) minutes and in the nonexposed groups 24(6) minutes (*P*=0.068).The onset time of sensory block in the four branches of brachial plexus block was also similar between the groups. The median (IQR) onset time of the sensory block in the nonexposed group and the exposed group of the ulnar nerve was 21(6) and 21(6) minutes (*P*=0.114), the radial nerve 18(6) and 16.5 (9) minutes (*P*=0.115), the musculocutaneous 24(6) and 24(3) minutes (*P*=0.165), and the median nerve 18(5) and 18(6) minutes (*P*=0.126), respectively. The time taken to start the incision (incision time) was also comparable between the groups (*P*=0.203).

Previous studies on the addition of dexamethasone to local anesthetics in brachial plexus block have yielded conflicting results regarding the onset time of sensory block. Similar to this study, there was no significant difference in the onset of sensory blockade of the axillary block between groups when dexamethasone was used^3,10^.

On the contrary, another research showed a significantly more rapid sensory block in the dexamethasone group than in the control group^17^ as well as prolonged sensory blockade^18^. The possible explanation for this difference might be due to different brachial plexus block techniques, study design effects, and local anesthetic concentration and dose differences.

In the current study, the duration of sensory block of the axillary brachial plexus was significantly longer (235 ±37) in the exposed group compared to (172±28) minutes in the nonexposed group, with a statistically significant difference. Similar results were observed in other studies where dexamethasone with lidocaine caused significantly prolonged sensory blockade in axillary brachial blocks^10,19^.

In this study, the first analgesia request time was analyzed by Kaplan–Meier analysis with a log-rank test and showed a significant difference in survival time from the first analgesic request between the groups (log-rank *P*-value <0.001). The median (IQR) time to the first analgesic request was 210 (185 to 230) minutes in the nonexposed group and 270 (240 to 330) minutes in the exposed group.

Similar to this study, a randomized controlled trial showed that the perineural addition of 2.5, 5, or 7.5 mg of dexamethasone to 0.5% ropivacaine for interscalene block increased the first analgesic request time in a dose-dependent manner^20^. Analgesic needs for 48 h were also reduced by perineural and intravenous dexamethasone^21^.

The severity of postoperative pain assessed for eight hours at 2, 4, 6, and 8 h starting from the incision using NRS showed an insignificant difference in the severity of pain at 2 h after the incision. This might be due to the fact that the majority of patients in both groups had not recovered from analgesia at the second hour after the incision. At 4 and 8 h, there was a significant difference in pain score between the groups, which was comparable with other studies^17^. At the sixth hour, the pain score was also not significantly different between the groups. This could be due to majority of patients took their first analgesic in the nonexposed group rather than the exposed group at this time.

There is debate over whether perineural corticosteroids may be harmful. Reports of neurotoxicity seem to be related to the vehicle polyethylene glycol and the preservative benzyl alcohol in some preparations, as well as the presence of insoluble steroid particulate matter in the injectate^22^. In our study, we used nonparticulate Dexamethasone, which is available in a preservative-free formulation.

### Strength

The study participants were homogenous and we used exclusion criteria to prevent confounding.

### Limitations

The study had certain limitations, including the variability of surgical procedures that were difficult to categorize based on the cutaneous innervation of brachial nerves. In trans-arterial techniques, doses of local anesthetics administered may not distribute equally to all four nerves.

### Relevance and implications

It is clinically relevant because dexamethasone is easily available in resource-limited countries; managing postoperative pain in ambulatory orthopedic surgery can avoid unnecessary hospital admissions, facilitate early recovery, reduce hospital stay time, and also increase patient satisfaction. It helps to reduce opioid-related side effects. It has implications for further research.

## Conclusions and recommendations

Our analysis showed that the addition of 8 mg dexamethasone to 1% lidocaine with adrenaline solution in trans-arterial axillary brachial plexus block for ambulatory elective hand, wrist, and forearm surgeries prolonged the duration of sensory blockade and analgesic request time but did not reduce the onset time of sensory block.

We recommend the addition of 8 mg dexamethasone to 1% lidocaine with adrenaline solution to prolong the duration of sensory block and the first analgesic request time. We also recommend randomized controlled clinical trials to compare the efficacy of 4 mg versus 8 mg dexamethasone and further research on its effect on nervous tissue and the risk of persistent nerve injury.

## Ethical approval

Ethical clearance and permission was obtained from Addis Ababa University college of health science institutional review board before the start of the study with reference no. 21/17/Ans.

## Consent

Informed written consent was obtained from every participant.

## Sources of funding

This work was funded by Addis Ababa University. The sponsor has no role in collection, analysis and interpretation of data; in the writing of the manuscript; and in the decision to submit the manuscript for publication.

## Author contribution

S.M.: conceptualization, methodology, data collection, formal analysis and interpretation, original manuscript development and revision; B.G.: conceptualization, data analysis and interpretation of results, manuscript development and revision. Both authors read and approved the final manuscript.

## Conflicts of interest disclosure

The authors declare no conflicts of interest.

## Research registration unique identifying number (UIN)

1. Name of the registry: Research Registry.
2. Unique identifying number: researchregistry8801.
3. Hyperlink to your specific registration (must be publicly accessible and will be checked): https://www.researchregistry.com/browse-the-registry#home/.

## Guarantor

Betelihem Girma and Simeneh Mola.

## Data availability statement

The data sets used and analyzed during the study are available upon reasonable requests.

## Provenance and peer review

Not commissioned, externally peer reviewed.
